# Suicide Risk and Associated Factors in Patients with Pancreatic Cancer: A Systematic Review

**DOI:** 10.3390/diseases14090346

**Published:** 2026-09-18

**Authors:** Maxence Chicoisneau, Christelle Bouchart, Matthieu Hein

**Affiliations:** 1 Faculté de Médecine, Université Libre de Bruxelles, ULB, 1070 Brussels, Belgium; maxence.chicoisneau@ulb.be; 2 Institut Jules Bordet, Hopital Universitaire de Bruxelles (H.U.B.), Department of Radiotherapy-Oncology, Université Libre de Bruxelles, ULB, 1070 Brussels, Belgium; christelle.bouchart@hubruxelles.be; 3 Laboratory of Experimental Gastroenterology, Université Libre de Bruxelles, ULB, 1070 Brussels, Belgium; 4 CHU Brugmann, Service de Psychiatrie et Laboratoire du Sommeil, Université Libre de Bruxelles, ULB, 1020 Brussels, Belgium; 5 Laboratoire de Psychologie Médicale et Addictologie (ULB312), Université Libre de Bruxelles, ULB, 1020 Brussels, Belgium

**Keywords:** pancreatic cancer, suicide, mortality, Epidemiology, psycho-oncology

## Abstract

Background/Objectives: Pancreatic cancer (PC) is characterized by poor survival and a substantial psychological burden. Growing evidence suggests that individuals diagnosed with PC are at an increased risk of suicide. This systematic review aimed to synthesize the available evidence on suicide risk among patients with PC and to identify factors associated with increased vulnerability. Methods: Following the PRISMA 2020 guidelines, a systematic review of the PubMed/MEDLINE and Scopus databases was conducted in August 2026. Of the 1015 records initially identified, 23 studies published between 2010 and July 2026 were included. Eligible studies specifically examined suicide risk among patients with PC using registry-based, administrative, or death certificate data. Risk of bias was assessed using the ROBINS-E tool. Results: Most included studies reported a higher suicide risk among patients with PC compared with the general population and, in many analyses, compared with patients with other cancer types. Large population-based studies generally reported standardized mortality ratios of approximately 2 to 11, although substantially higher estimates were observed in selected populations and specific subgroups. Suicide risk was particularly elevated during the early post-diagnosis period (with several studies identifying the greatest excess risk within the first two months following diagnosis), remained elevated throughout the first year and gradually declined thereafter. Male sex emerged as the most consistently demonstrated risk factor for suicide, while associations with unmarried status were observed across several studies but were derived largely from partially overlapping SEER-based cohorts. Additional evidence suggests a potential role for social vulnerability and poor prognostic factors. Conclusions: Despite the predominantly observational nature of the available evidence, the consistency of the findings highlights the importance of increased awareness of suicide vulnerability among patients with PC and supports consideration of psychosocial assessment and mental health support within routine pancreatic cancer care. Future prospective studies are needed to better characterize high-risk patients and to inform targeted suicide prevention strategies.

## 1. Introduction

Pancreatic cancer (PC) represents a major public health challenge due to its increasing incidence and particularly poor prognosis. It is currently the third leading cause of cancer-related death in the United States and ranks seventh worldwide among cancer-related causes of mortality [1]. Despite therapeutic advances, overall survival remains limited, with a 5-year survival rate of approximately 12%, one of the lowest among all cancer types [2]. This poor prognosis is largely attributable to late-stage diagnosis, as nearly 50% of patients present with metastatic disease at the time of diagnosis, while only 10–15% have localized disease that is amenable to potentially curative surgical resection [3]. Furthermore, the incidence of PC continues to rise at an estimated annual rate of 0.5–1.0%, and projections suggest that it may become the second leading cause of cancer-related death by 2030 [4].

Beyond its adverse impact on survival, PC is associated with a substantial psychological burden. The prevalence of depression among patients with PC is estimated at 31% (95% CI: 20–42%), while anxiety affects approximately 20% of patients (95% CI: 9–32%), both rates exceeding those reported for many other cancer types [5]. Notably, depressive symptoms may precede the diagnosis of PC, suggesting a potential paraneoplastic phenomenon mediated by tumor-related inflammatory and metabolic alterations [6]. Consistent with these observations, the National Comprehensive Cancer Network (NCCN) identifies PC as one of the malignancies associated with the highest levels of psychological distress and depressive symptoms, underscoring the importance of systematic psychosocial assessment and supportive care in this population [7].

In this context, increasing attention has been directed toward suicide risk among patients with PC. Population-based studies have consistently shown that patients with PC experience some of the highest suicide rates among all cancer types [8,9]. For example, an analysis of the Surveillance, Epidemiology, and End Results (SEER) database including 199,604 patients diagnosed between 2000 and 2018 reported a suicide rate of 88.05 per 100,000 person-years, corresponding to a standardized mortality ratio (SMR) of 6.43 compared with the general population [6]. Similarly, other studies have reported SMRs ranging from 3.89 to 10.80, consistently demonstrating a substantially elevated risk relative to both the general population and most other malignancies [9,10,11]. Notably, this excess risk appears to be particularly pronounced during the first six months following diagnosis and remains significantly higher than that observed in the general population for several years thereafter [8,12,13,14]. Furthermore, several factors associated with suicide risk have been identified among patients with PC [9,11]. Male sex and unmarried status have been associated with an increased suicide risk, whereas marital status, receipt of chemotherapy, and surgical treatment appear to favor a lower suicide risk. However, some evidence suggests a transient increase in suicide risk during the early postoperative period, highlighting the complex relationship between cancer treatment and psychological vulnerability [9,11].

Despite these concerning findings, no systematic review has, to our knowledge, specifically synthesized the available evidence on suicide risk and associated factors among patients with PC. Therefore, the primary objective of this systematic review was to evaluate and quantify the excess suicide risk in patients with PC. In addition, based on the studies included in this synthesis, the secondary objective was to identify factors associated with suicide in this population. A better understanding of these factors may facilitate risk stratification, inform psychosocial screening, optimize clinical management, and support the development of targeted suicide prevention strategies for patients with PC.

## 2. Methods

### 2.1. Article Selection

In accordance with the PRISMA (Preferred Reporting Items for Systematic Reviews and Meta-Analyses) 2020 recommendations, a systematic review was conducted to investigate suicide risk in individuals with PC. The initial review protocol before revisions was registered in PROSPERO (CRD420261441778). The literature search and study were conducted between 17 August 2026 and 23 August 2026 using the PubMed-Medline and Scopus databases. The search strategy used the following keyword algorithm: (“Pancreatic Neoplasms”[Mesh] OR Pancreatic Cancer OR Pancreatic Adenocarcinoma OR Pancreatic Ductal Adenocarcinoma OR Pancreas) AND (“Suicide”[Mesh] OR Suicide OR Suicidal Behavior OR Self-harm OR Suicidal Death OR Suicide Mortality OR Suicidal Mortality) for the PubMed-Medline database and (TITLE-ABS-KEY (“Pancreatic Neoplasms” OR “Pancreatic Cancer” OR “Pancreatic Adenocarcinoma” or “Pancreatic Ductal Adenocarcinoma” OR “Pancreas”) AND TITLE-ABS-KEY (“Suicide” OR “Suicidal Behavior” OR “Self-harm” OR “Suicidal Death” OR “Suicide Mortality” OR “Suicidal Mortality”)) for the Scopus database.

After applying this keyword algorithm, this search identified 1015 articles, which were independently evaluated by two reviewers. The inclusion and exclusion criteria applied for the selection of articles were:

- Articles with available analyses specifically targeting the suicide risk associated with PC (either exclusively in PC cohorts or through separate PC-specific analyses when multiple cancer types were included).
- Deaths by suicide or probable suicide identified from reliable national or administrative databases, based on ICD codes or national judicial procedures.
- Diagnosis of PC confirmed by clinical diagnosis or by diagnostic codes from international classification systems.
- All types of study designs (cross-sectional, longitudinal, prospective, retrospective, interventional, and experimental), with the exception of literature reviews, book chapters, case reports, opinion articles, animal studies, letters to the editor and conference papers.
- Articles published between 1 January 2010 and 31 July 2026.
- Articles written in English or French.
- Articles available in full text.

**Figure 1:**
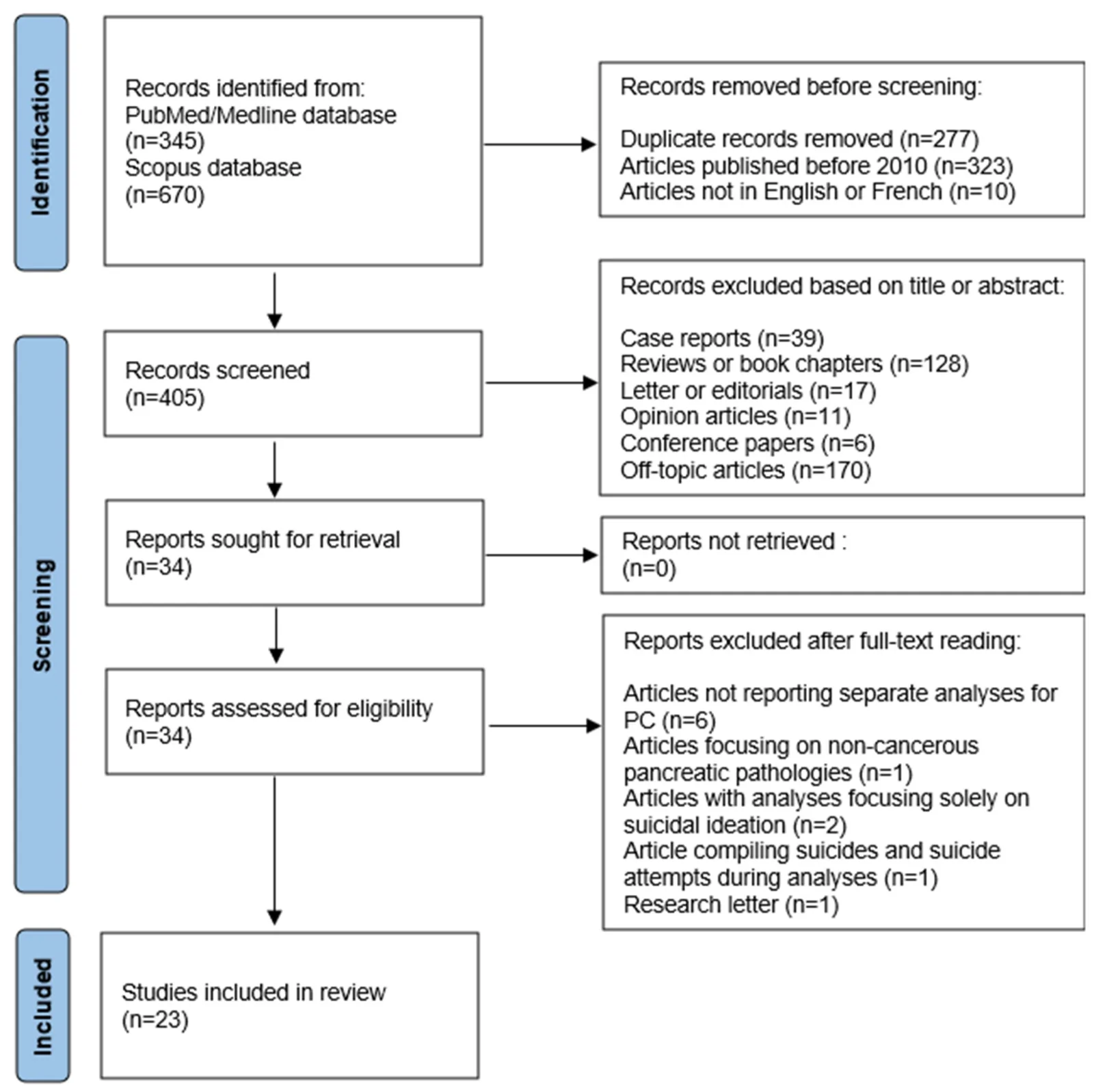
Identification of studies via databases and registers.

After applying these inclusion and exclusion criteria, 23 articles specifically investigating suicide risk in patients with PC met the eligibility criteria and were included in the final review (Figure 1) [8,9,10,11,12,13,15,16,17,18,19,20,21,22,23,24,25,26,27,28,29,30,31]. Any disagreement regarding the selection of articles was discussed and resolved by the two reviewers.

### 2.2. Quality Assessment and Risk of Bias Evaluation

The quality of the studies included in this systematic review was independently assessed by two reviewers using the methodological guidelines developed by the Agence Nationale d’Accréditation et d’Évaluation en Santé (ANAES), integrated into the Haute Autorité de Santé (HAS) [32]. According to these guidelines, three grades of recommendation may be assigned based on four levels of scientific evidence (Supplementary Data S1). In addition, the risk of bias for each study was independently evaluated by the two reviewers using the ROBINS-E tool (Risk Of Bias In Non-randomized Studies—of Exposures), which assesses bias across seven specific domains [33]. Any discrepancies in quality assessment were resolved by consensus.

### 2.3. Data Extraction

Following independent work by the two reviewers, the data extracted from each of the 23 articles selected for this systematic review of the literature were:

1. Study characteristics: first author’s name, year of publication, country of study, study design, evidence level, recommendation grade, population size, recruitment period and main limitations.
2. Patient characteristics: age, sex, and ethnicity, when available, as well as overall population features and reported clinical characteristics.
3. Clinical and methodological data: main inclusion and exclusion criteria, stage of PC and treatment modalities, when specified.
4. Exposure and outcome assessment: number of suicide events, timing of assessment and method of suicide ascertainment.
5. Suicide-related outcomes in patients with PC: suicide rates, SMR, O/E ratios (observed-to-expected ratios), aIRR (adjusted incidence rate ratios) and AER (absolute excess risk), with 95% confidence intervals; time-, stage-, or sex-stratified analyses; survival outcomes; and multivariate analyses reporting HR or OR (odds ratios) for factors associated with suicide risk.

Any disagreements regarding data extraction were discussed and resolved by both reviewers. All relevant data extracted from the included studies are provided in the manuscript and tables.

## 3. Results

### 3.1. Characteristics of Studies and Identification of Suicides (Table 1)

All studies investigating suicide risk following a diagnosis of PC were observational in design [8,9,10,11,12,13,15,16,17,18,19,20,21,22,23,24,25,26,27,28,29,30,31]. The vast majority consisted of retrospective population-based cohort studies based on national cancer registries, administrative databases, or registry-linked datasets [8,9,10,11,12,13,15,16,17,18,19,20,21,22,23,24,25,26,27,28,29,30,31]. One study employed a population-based matched cohort design [29], whereas another combined data from a national cancer registry with a single-center palliative care cohort [23]. Consequently, all included studies were classified as providing a low level of evidence (Level 4—Grade C) [8,9,10,11,12,13,15,16,17,18,19,20,21,22,23,24,25,26,27,28,29,30,31].

Overall, most of the available evidence originated from the United States [9,11,12,13,16,17,19,20,25,26,27,30,31]. Several studies were based on the Surveillance, Epidemiology, and End Results (SEER) database, which constituted the principal source of evidence and accounted for a substantial proportion of the included participants [9,11,13,17,19,25,26,27,30,31]. Additional United States-based evidence was derived from the California Cancer Registry [16], the North American Association of Central Cancer Registries [12], and the Veterans Health Administration database [20]. Evidence from Asia was primarily generated in South Korea using nationwide resources such as the Korea Central Cancer Registry and the National Health Insurance Service database [15,18], with additional data from Taiwan obtained from the Taiwan Cancer Registry [22]. European evidence was more geographically diverse but comparatively limited, including studies conducted in England [10,29], Denmark [8], Austria [23], Lithuania [24], and Poland [28].

When reported, the characteristics of PC cohorts generally reflected an older population, with most patients aged 60 years or older [9,11,21,28,29,30]. Men accounted for approximately half of the study populations and represented the majority of participants in most cohorts [9,11,21,28,29,30], although their proportion was substantially higher in the Veterans Health Administration cohort because of its predominantly veteran population [20]. Ethnicity was reported mainly in United States-based studies and consistently demonstrated a predominance of White patients, who generally accounted for approximately three-quarters to four-fifths of included participants [9,11,21,30]. However, detailed clinical characteristics were inconsistently reported across studies. Tumor stage was available only in a subset of investigations and generally indicated that most patients presented with advanced disease at the time of diagnosis [9,11,13,21,29,30,31]. For example, distant-stage disease accounted for more than half of cases in several SEER-based cohorts [9,11,13,30]. Information regarding cancer treatment was also limited. Although some studies reported data on surgery, chemotherapy, and radiotherapy [9,11,21,27,30], many registry-based investigations lacked treatment-specific information, precluding comprehensive comparisons across cohorts [10,15,16,17,18,19,20,22,23,24,25,26,28,29,31].

The ascertainment of suicide outcomes relied almost exclusively on cause-of-death classifications obtained from cancer registries, national mortality registries, or administrative databases [8,9,10,11,12,13,15,16,17,18,19,20,21,22,23,24,25,26,27,28,29,30,31]. In most studies, suicide was identified using ICD-9 or ICD-10 codes for intentional self-harm, registry-based mortality records, or SEER cause-of-death classifications [8,9,10,11,12,13,15,16,17,18,19,20,21,22,23,24,25,26,27,28,29,30,31]. Suicide outcomes were generally evaluated during follow-up after the diagnosis of PC [8,9,10,11,12,13,15,16,17,18,19,20,21,22,23,24,25,26,27,28,29,30,31], and several studies conducted stratified analyses according to time since diagnosis [8,13,15,17,21,22,28,30]. Nevertheless, outcome definitions were not entirely consistent across studies. For example, the English registry study included deaths of undetermined intent (open verdicts) in addition to confirmed suicides [10], whereas the Austrian study supplemented registry-based classifications with information obtained from autopsy reports to identify potential suicide cases [23].

**Table 1:** Characteristics of the selected studies.

Studies	Country of Study Study Design Evidence Level Recommendation Level	Population and Recruitment Period	Patient Characteristics	Main Inclusion and Exclusion Criteria	Stage of PC	Treatment of PC	Suicide Assessment	Suicide Outcome
Ahn et al., (2010) [15]	South Korea Retrospective population-based cohort (Korea Central Cancer Registry) Level 4—Grade C	18,632 patients with PC (2.3% of the whole sample) 1993–2005	Data only available for the whole sample (816,295 patients with cancers): 47.2% ≥60 years 54.7% male	Inclusion: patients registered in national cancer registry Exclusion: multiple primary cancers, missing data, inconsistent dates	Not specified for the PC subgroup	Not specified for the PC subgroup	Time of assessment: follow-up after diagnosis (up to >5 years) Measurement: death records (ICD-10 codes: suicide/self-inflicted injury)	Suicide rate in PC Comparison with general population using SMR Sex- and time-stratified analyses
Turaga et al., (2011) [11]	United States Retrospective population-based cohort (SEER database) Level 4—Grade C	36,221 patients with PC 1992–2005	68.5 years (IQR: 60–77) 50.8% male 81.9% white	Inclusion criteria: diagnosis of PC Exclusion criteria: other malignant neoplasms	Localized: 7.0% Regional: 31.7% Distant: 61.3%	16.5% surgery 20.4% radiotherapy Chemotherapy (not available)	Time of assessment: during follow-up after diagnosis Measurement: cause of death (SEER codes: suicide/self-inflicted injury)	Suicide rate Comparison with general population using SMR Survival comparison between suicide vs. non-suicide patients Multivariate analyses to identify suicide risk factors
Nasseri et al., (2012) [16]	United States Retrospective population-based cohort (California Cancer Registry) Level 4—Grade C	1,123,528 patients with cancers (not specified for the PC subgroup) 1997–2006	Data only available for the whole sample: 49.7% male 62.0% white	Inclusion: cancers diagnosed between 1997 and 2006 with known age and sex Exclusion: multiple primary cancers, cases identified only by autopsy or death certificate.	Not specified for the PC subgroup	Not specified for the PC subgroup	Time of assessment: during follow-up after diagnosis Measurement: suicide identified by ICD-10 codes	Comparison with general population using suicide rate ratio and age-adjusted rate ratio Sex- and race-stratified analyses
Kaceniene et al., (2017) [24]	Lithuania Population-based retrospective cohort (Lithuanian Cancer Registry) Level 4—Grade C	273,511 patients with cancers (not specified for the PC subgroup) 1993–2012	Data only available for the whole sample: 50.9% male	Inclusion: first primary cancer diagnosed between 1993 and 2012 Exclusion: cancer diagnosis on the date of death, lost to follow-up, missing cause-of-death information	Not specified for the PC subgroup	Not specified for the PC subgroup	Time of assessment: during follow-up after diagnosis Measurement: suicide identified by ICD-9 and ICD-10 codes	Comparison with general population using SMR Sex-stratified analyses
Anderson et al., (2018) [17]	United States Retrospective population-based cohort (SEER database) Level 4—Grade C	119,194 patients with PC (14.0% of the whole sample) 2000–2014	Data only available for the whole sample (856,293 patients with digestive system cancers): 69% ≥60 years 54% male 78% white	Inclusion: primary digestive system cancers with known age Exclusion: cases identified by death certificate or autopsy only	Not specified for the PC subgroup	Not specified for the PC subgroup	Time of assessment: during follow-up after diagnosis Measurement: cause of death (SEER codes: suicide/self-inflicted injury)	Suicide rate in PC Comparison with general population using SMR Time-stratified analyses
Saad et al., (2019) [13]	United States Retrospective population-based cohort (SEER database) Level 4—Grade C	119,194 patients with PC (2.6% of the whole sample) 2000–2014	Data only available for the whole sample (4,671,989 patients with digestive system cancers): 49.7% ≥65 years 51.4% male 82.3% white	Inclusion: histologically confirmed cancers diagnosed 2000–2014 Exclusion: not specified	Localized or regional: 36.7% Distant: 52.8% Unknown: 10.5%	Not specified for the PC subgroup	Time of assessment: within 1 year after diagnosis Measurement: cause of death (SEER codes: suicide/self-inflicted injury)	Suicide within 1 year after diagnosis Observed vs. expected comparison Stage- and time-stratified analyses
Henson et al., (2019) [10]	England Retrospective population-based cohort (Public Health England’s national cancer registration database) Level 4—Grade C	121,207 patients with PC (2.6% of the whole sample) 1995–2015 (follow-up until 2017)	Data only available for the whole sample (4,722,099 patients with cancers): 74.3% ≥60 years 50.3% male 65.4% white	Inclusion: adults with malignant cancer Exclusion: non-melanoma skin cancer; diagnosis at death only	Not specified for the PC subgroup	Not specified for the PC subgroup	Time of assessment: follow-up after diagnosis Measurement: death records (ICD-9 or ICD-10 codes: suicide/open verdicts)	Comparison with general population using SMR and AER per 10,000 Sex-stratified analyses
Abdel-Rahman, (2019) [25]	United States Retrospective population-based cohort (SEER database) Level 4—Grade C	62,080 patients with PC (2.0% of the whole sample) 2000–2010 (follow-up until 2015)	Data only available for the whole sample (3,149,235 patients with cancers): 35.4% ≥70 years 51.9% male 82.9% white	Inclusion: adults with malignant cancer Exclusion: missing data	Not specified for the PC subgroup	Not specified for the PC subgroup	Time of assessment: follow-up after diagnosis Measurement: suicides identified through SEER mortality data	Observed vs. expected comparison
Choi et al., (2021) [18]	South Korea Retrospective population-based cohort (NHIS national cohort) Level 4—Grade C	972 patients with PC 2005–2013	Data only available for the whole sample of patients with cancers: 51.6% ≥60 years 49.2% male	Inclusion: adults in national insurance cohort Exclusion: diagnosed cancer between 2002 and 2004, missing data	Not specified for the PC subgroup	Not specified for the PC subgroup	Time of assessment: follow-up after diagnosis Measurement: death records (ICD-10 codes: suicide/self-inflicted injury)	Cox regression adjusted for confounders
Ma et al., (2022) [9]	United States Retrospective population-based cohort (SEER database) Level 4—Grade C	199,604 patients with PC 2000–2018	Mean age 69.7 years 50.5% male 80.2% white	Inclusion: primary malignant pancreatic tumors Exclusion: unknown age, race, follow-up time, only autopsy diagnosis	Localized: 10.7% Regional: 28.6% Distant: 51.2% Unknown: 9.3%	18.0% surgery 13.4% radiotherapy 43.3% chemotherapy	Time of assessment: during follow-up after diagnosis Measurement: cause of death (SEER codes: suicide/self-inflicted injury)	Suicide rate Comparison with general population using SMR Time-stratified analyses Multivariate analyses to identify suicide risk factors
Su et al., (2022) [26]	United States Retrospective population-based cohort (SEER database) Level 4—Grade C	16,815 patients with PC (2.6% of the whole sample) 1975–2016	Data only available for the whole sample (645,818 patients with multiple primary cancers): 79.9% ≥60 years 54.0% male 84.6% white	Inclusion: patients with one or more primary cancers Exclusion: patients with cancers diagnosed only by autopsy reports or death certificates, incomplete follow-up data.	Not specified for the PC subgroup	Not specified for the PC subgroup	Time of assessment: during follow-up after diagnosis Measurement: suicide identified by ICD-8, ICD-9, ICD-10 codes	Suicide rate Comparison with general population using SMR
Katayama et al., (2023) [19]	United States Retrospective population-based cohort (SEER database) Level 4—Grade C	68,818 patients with PC (18.0% of the whole sample) 2004–2016	Data only available for the whole sample (382,266 patients with gastrointestinal cancers): 72.0 years (IQR: 65.0–80.0) 52.4% male 74.4% white	Inclusion: Medicare beneficiaries with gastrointestinal cancers Exclusion: incomplete Medicare coverage or participation in a health maintenance organization	Not specified for the PC subgroup	Not specified for the PC subgroup	Time of assessment: during follow-up after diagnosis Measurement: cause of death (SEER codes: suicide/self-inflicted injury)	Suicide rate in PC
Hu et al. (2023) [12]	United States Retrospective population-based cohort (North American Association of Central Cancer Registries) Level 4—Grade C	425,278 patients with PC (2.5% of the whole sample) 2000–2016	Data only available for the whole sample (16,771,397 patients with cancers): 50.9% ≥65 years 51.5% male 78.4% white	Inclusion: first primary malignant cancer diagnosed between 2000 and 2016 Exclusion: cancers reported by death certificate or autopsy only, incomplete follow-up data	Not specified for the PC subgroup	Not specified for the PC subgroup	Time of assessment: during follow-up after diagnosis Measurement: suicide identified by ICD-10 codes	Comparison with general population using SMR Multivariable Cox regression to identify cancer-specific risk factors for suicide Time-stratified analyses
Potter et al. (2023) [27]	United States Retrospective population-based cohort (SEER database) Level 4—Grade C	23,770 patients with PC (1.3% of the whole sample) 2000–2016	Data only available for the whole sample (1,811,397 patients with cancers): 62 years (IQR: 52–72) 25.6% male 81.1% white	Inclusion: patients undergoing cancer-directed surgery for one of 15 major solid malignancies Exclusion: multiple primary cancers	Not specified for the PC subgroup	Surgical treatment for all patients Chemotherapy and radiotherapy data not specified for the PC subgroup	Time of assessment: during follow-up after diagnosis Measurement: death certificate (SEER codes: suicide/self-inflicted injury)	Comparison with general population using SMR Timing of suicide after surgery
Michalek et al. (2023) [28]	Poland Retrospective population-based cohort (Polish National Cancer Registry) Level 4—Grade C	33,440 patients with PC (2.3% of the whole sample) 2009–2019	Patients with PC: Mean age 66.5 (11.4) years 49.3% male	Inclusion: patients aged ≥15 years diagnosed with a primary malignant neoplasm Exclusion: non-melanoma skin cancers, duplicate primary tumors	Not specified for the PC subgroup	Not specified for the PC subgroup	Time of assessment: during follow-up after diagnosis Measurement: suicides identified through the national death registry using ICD-10	Comparison with general population using SMR Sex and time-stratified analyses
Forbes et al. (2024) [29]	United Kingdom Population-based matched cohort study (UK Clinical Practice Research Datalink) Level 4—Grade C	24,224 patients diagnosed with PC compared with 231,873 matched controls without cancer 1998–2018	Patients with PC: 85.1% ≥60 years 49.9% male	Inclusion: adults ≥18 years with a first diagnosis of one of the 20 most common cancers in the UK Exclusion: prior cancer (except non-melanoma skin cancer), incomplete data	Early: 9.7% Late: 35.3% Missing: 55.0%	Not specified for the PC subgroup	Time of assessment: during follow-up after diagnosis Measurement: suicide identified by ICD-9 and ICD-10 codes	Incidence rate Cox regression models compared suicide risk in cancer survivors with matched cancer-free individuals
Wang et al. (2024) [30]	United States Retrospective population-based cohort (SEER database) Level 4—Grade C	101,382 patients with PC 2000–2017	63.12% ≥65 years 51.13% male 80.49% white	Inclusion: histologically confirmed PC Exclusion: cancer diagnosis by autopsy or death certificate only, incomplete data	Localized: 8.3% Regional: 37.2% Distant: 51.1% Unknown: 3.5%	21.08% surgery 20.00% radiotherapy 60.10% chemotherapy	Time of assessment: during follow-up after diagnosis Measurement: cause of death (SEER codes: suicide/self-inflicted injury)	Suicide rate Comparison with general population using SMR Multivariable Cox regression to identify cancer-specific risk factors for suicide Age- and time-stratified analyses
Kinslow et al. (2024) [31]	United States Retrospective population-based cohort (SEER database) Level 4—Grade C	179,063 patients with PC (2.7% of the whole sample) 2000–2019	Data only available for the whole sample (6,754,704 patients with cancers): 52.8% ≥65 years 50.5% male 82.5% white	Inclusion: patients aged ≥16 years newly diagnosed with any malignant tumor Exclusion: cancer diagnosis by autopsy or death certificate only, incomplete data	Not specified for the PC subgroup	Not specified for the PC subgroup	Time of assessment: during follow-up after diagnosis Measurement: suicide identified by death certificate records	Suicide rate within 6 months after cancer diagnosis Comparison with general population using SMR for completed suicide within 6 months after cancer diagnosis Stage-stratified analyses
Kittel et al., (2025) [20]	United States Retrospective population-based cohort (Veterans Health Administration database) Level 4—Grade C	9619 patients with PC (2.19% of the whole sample) 2010–2020	Data only available for the whole sample (439,667 patients with cancers): 38.5% ≥70 years 96.6% male 73.9% white	Inclusion: veterans diagnosed with malignant cancer and treated in the Veterans Health Administration Exclusion: invalid dates, diagnosis at autopsy, age > 110, incomplete records	Not specified for the PC subgroup	Not specified for the PC subgroup	Time of assessment: throughout follow-up after diagnosis and treatment Measurement: death records (ICD-10 codes: suicide/self-inflicted injury)	Suicide rate in PC
Fitzgerald et al., (2025) [8]	Denmark Retrospective population-based cohort (national registers) Level 4—Grade C	707,513 patients with cancers (not specified for the PC subgroup) 2000–2021	Data only available for the whole sample (707,513 patients with cancers): 69 years (IQR: 59–77) 47% male	Inclusion: individuals ≥ 15 years in national registers Exclusion: prior history of cancer, diagnosis of a second cancer during follow-up, non-melanoma skin cancer.	Not specified for the PC subgroup	Not specified for the PC subgroup	Time of assessment: up to 5 years post-diagnosis Measurement: death records (ICD-10 codes for suicide or suicide reported in the national death registry)	Comparison with general population using aIRR
Gao et al., (2026) [21]	United States Retrospective population-based cohort (SEER database) Level 4—Grade C	9624 patients with PC 2010–2021	66 years (IQR: 20–89) 51.2% male 80.1% white	Inclusion: adults with PC receiving chemotherapy Exclusion: missing key clinical variables, incomplete follow-up	Stage I/II: 38.4% Stage III/IV: 61.6%	53.1% surgery 20.7% radiotherapy 100% Chemotherapy	Time of assessment: follow-up after diagnosis Measurement: death records (ICD-10 codes: suicide/self-inflicted injury)	Comparison with general population using SMR Time-stratified analyses
Hsiao et al., (2026) [22]	Taiwan Population-based retrospective cohort (Taiwan Cancer Registry) Level 4—Grade C	Nationwide cohort (total number of subjects and for the PC group not specified) 1985–2018 (follow-up to 2020)	Data only available for the whole sample: Age ≥45 years	Inclusion: adults ≥45 years with invasive cancers Exclusion: incomplete data, non-melanoma skin cancers	Not specified for the PC subgroup	Not specified for the PC subgroup	Time of assessment: follow-up after diagnosis Measurement: death records (ICD-9 or ICD-10 codes for suicide)	Suicide rate in PC Time-stratified analyses
Listabarth et al., (2026) [23]	Austria Retrospective population-based cohort (Austrian Cancer Registry + single-center cohort) Level 4—Grade C	28,810 patients with PC (181 in palliative care + 28,629 in control group) 2012–2022	Palliative care 75.6 years (IQR: 66.0–83.2) 44.6% male Control Group 66.2 years (IQR: 55.5–74.1) 53.3% male	Palliative care: oncological patients admitted to palliative care ward Control: national cancer registry (diagnosis-matched) No major exclusion except data availability	Not specified for the PC subgroup	Not specified for the PC subgroup	Time of assessment: follow-up after diagnosis Measurement: death records (ICD-10 codes for suicide, potential suicide via autopsy reports)	Cumulative incidence for suicide

### 3.2. Impact of PC Diagnosis on Suicide Risk

#### 3.2.1. Increased Suicide Risk Associated with PC (Table 2)

Across all study settings, findings were broadly convergent: a diagnosis of PC was associated with a markedly increased suicide risk compared with the general population.

Findings from studies conducted in the United States consistently demonstrated an elevated suicide risk among patients with PC, although the magnitude of the association varied according to cohort characteristics and analytical approaches [9,11,12,13,16,17,19,20,25,26,27,30,31]. Although exceptionally high SMR estimates were occasionally observed in selected subpopulations (SMR 113.68) [21], large population-based studies generally reported SMRs ranging from 2.50 to 10.80 [9,11,12,17,25,26,30,31]. Turaga et al. (2011) reported a suicide rate of 135.5 per 100,000 person-years and an SMR of 10.8 (95% CI: 9.2–12.7), corresponding to more than a tenfold increase compared with the general population [11]. Similarly, Ma et al. (2022) identified 180 suicides among 199,604 patients with PC, yielding a suicide rate of 88.05 per 100,000 person-years and an SMR of 6.43 (95% CI: 5.49–7.37) [9]. Anderson et al. (2018) identified PC as the gastrointestinal malignancy associated with the highest suicide risk, reporting an SMR of 5.28 (95% CI: 4.26–6.47) [17]. Consistent findings were reported by Nasseri et al. (2012), who observed significantly elevated suicide rate ratios among male and White patients with PC [16], and by Abdel-Rahman (2019), who reported an O/E ratio of 3.70 (95% CI: 2.70–4.95), with socioeconomic deprivation associated with a higher risk of suicide [25]. Additional analyses reported SMRs of 5.98 (95% CI: 3.81–9.37) among patients with multiple primary cancers [26], 3.51 (95% CI: 2.81–4.32) in a recent SEER-based cohort [30], and 9.7 (95% CI: 7.8–11.8) during the first six months following diagnosis [31]. Furthermore, Hu et al. (2023) reported an overall SMR of 2.50 (95% CI: 2.24–2.76) and demonstrated that PC was associated with a higher suicide risk than colorectal cancer during the first two years after diagnosis [12]. In the Veterans Health Administration cohort, suicide risk reached 173.9 per 100,000 person-years, among the highest rates reported across all cancer types [20].

Findings from Asian populations were similarly consistent [15,18,22]. In South Korea, Ahn et al. (2010) reported elevated suicide risk among both men (SMR 6.01 [95% CI: 4.33–8.33]) and women (SMR 2.76 [95% CI: 1.15–6.63]), with PC exhibiting the highest suicide risk among cancers in men [15]. Choi et al. (2021) confirmed these findings and reported a significantly increased suicide risk among men with PC (HR 3.78 [95% CI: 1.21–11.78]), whereas no statistically significant association was observed among women [18]. Data from Taiwan further indicated that PC was among the cancer types associated with the highest suicide risk. Importantly, suicides were concentrated during the first two years following diagnosis, after which the risk gradually declined, although it remained elevated compared with that of the general population [22].

European studies likewise consistently reported an increased suicide risk among patients with PC [8,10,23,24,28,29]. In England, Henson et al. (2019) reported an SMR of 3.89 (95% CI: 2.77–5.48), together with an absolute excess risk of 2.89 per 10,000 person-years [10]. In Denmark, Fitzgerald et al. (2025) identified PC as one of the malignancies associated with the greatest increase in suicide risk, reporting an aIRR of 7.4 (95% CI: 6.3–8.6) [8]. Additional registry-based studies from Lithuania and Poland reported SMRs of 2.34 (95% CI: 1.22–4.50) and 1.77 (95% CI: 0.85–3.25), respectively [24,28]. In the Polish cohort, the highest risk was observed during the first six months following diagnosis (SMR 2.66 [95% CI: 0.98–5.80]) [28]. Austrian data demonstrated a significantly higher cumulative incidence of suicide among patients with PC receiving palliative care than among matched controls [23]. Finally, a large matched cohort study conducted in the United Kingdom reported that patients with PC had an almost sevenfold higher suicide risk than cancer-free controls (adjusted HR 6.85, 95% CI: 2.37–19.78) [29].

**Table 2:** Main results of the selected studies.

Studies	Main Results for Patients with PC	Main Limitations
Ahn et al., (2010) [15]	Men 36 suicides Suicide rate: 321.5 per 100,000 person-years SMR: 6.01 (95% CI: 4.33–8.33) Time-stratified <1 year: SMR 6.56 (95% CI: 3.96–10.89)—543.6 suicides per 100,000 person-years 1–4 years: SMR 8.10 (95% CI: 5.17–12.70)—488.0 suicides per 100,000 person-years ≥5 years: SMR 1.47 (95% CI: 0.37–5.87)—44.0 suicides per 100,000 person-years Women 5 suicides Suicide rate: 57.6 per 100,000 person-years SMR: 2.76 (95% CI: 1.15–6.63) Time-stratified <1 year: SMR 7.61 (95% CI: 3.17–18.29)—241.9 suicides per 100,000 person-years	No data on psychiatric comorbidities, possible misclassification or underreporting of suicide, missing data for the PC subgroup, lack of psychosocial and clinical confounders, low number of suicide events, retrospective design
Turaga et al., (2011) [11]	30 suicides Suicide rate: 135.5 per 100,000 person-years SMR: 10.8 (95% CI: 9.2–12.7) Median overall survival: 2 months (IQR: 0.5–3) for those who committed suicide vs. 4 months for other patients (IQR: 4–5) Median survival after operative intervention: 2 months (IQR: 0.5–18) for those who committed suicide vs. 10 months for other patients (IQR: 5–19) Univariate analyses: male sex was associated with significantly increased suicide risk compared to female sex (OR 13.5 [95% CI 3.2–56.9], *p* < 0.001) Multivariate analyses (only for men): married status was associated with significantly decreased suicide risk (OR 0.3 [95% CI 0.1–0.6], *p* = 0.002), whereas having undergone surgery was associated with significantly increased suicide risk (OR 2.5 [95% CI 1.0–6.4], *p* = 0.05)	No data on psychiatric comorbidities, possible misclassification or underreporting of suicide, lack of chemotherapy data, limited adjustment for confounders, retrospective design with small number of suicide events, risk of population overlap with other SEER-based studies
Nasseri et al., (2012) [16]	22 suicides Sex-stratified Male: 22 suicides—suicide rate ratio 2.4 (*p* < 0.05)—age-adjusted rate ratio 62.3/100,000 person-years Female: 1 suicide—insufficient data Race-stratified White: 16 suicides—suicide rate ratio 2.3 (*p* < 0.05)—age-adjusted rate ratio 68.6/100,000 person-years	Lack of psychiatric data, risk of underreporting or misclassification of suicide, small number of events for some subgroups (including women with PC), missing data for the PC subgroup, retrospective design
Kaceniene et al., (2017) [24]	9 suicides SMR: 2.34 (95% CI: 1.22–4.50) Sex-stratified Male: 7 suicides—SMR 2.18 (95% CI: 1.04–4.57) Female: 2 suicides—SMR 3.19 (95% CI: 0.80–12.76)	Lack of psychiatric or psychosocial data, risk of underreporting or misclassification of suicide, small number of suicide events, missing data for the PC subgroup, retrospective design
Anderson et al., (2018) [17]	93 suicides Suicide rate: 86.04 per 100,000 person-years SMR: 5.28 (95% CI: 4.26–6.47) Highest suicide risk among digestive cancers Time-stratified <2 months: SMR 9.72 (95% CI: 6.35–14.25)—157.74 suicides per 100,000 person-years 2 months–1 year: SMR 7.31 (95% CI: 5.39–9.70)—118.92 suicides per 100,000 person-years 1–5 years: SMR 2.65 (95% CI: 1.54–4.24)—43.42 suicides per 100,000 person-years ≥5 years: SMR 1.02 (95% CI: 0.12–3.68)—16.53 suicides per 100,000 person-years	No data on psychiatric comorbidities, possible misclassification or underreporting of suicide, missing data for the PC subgroup, lack of psychosocial and clinical confounders, retrospective design, risk of population overlap with other SEER-based studies
Saad et al., (2019) [13]	74 suicides within 1 year after diagnosis O/E ratio: 8.01 (95% CI: 6.29–10.06) Time-stratified First 6 months: O/E 9.46 (95% CI: 7.18–12.23) Second 6 months: O/E 5.15 (95% CI: 2.94–8.36) After more than 1 year: O/E 2.27 (95% CI: 1.36–3.54) Stage-stratified Localized/regional: O/E 4.8 (95% CI: 3.01–7.27) Distant: O/E 11.61 (95% CI: 8.47–15.53)	No data on psychiatric comorbidities, possible misclassification or underreporting of suicide, missing data for the PC subgroup, lack of psychosocial and clinical confounders, retrospective design, main analyses limited to first year, risk of population overlap with other SEER-based studies
Henson et al., (2019) [10]	33 suicides SMR: 3.89 (95% CI: 2.77–5.48) AER: 2.89 (95% CI: 1.56–4.22) Men SMR: 3.99 (95% CI: 2.70–5.90) AER: 4.41 (95% CI: 2.10–6.72) Women SMR: 3.62 (95% CI: 1.81–7.23) AER: 1.37 (95% CI: 0.06–2.68) PC among highest suicide risk cancers	No adjustment for psychiatric comorbidities, possible underreporting or misclassification of suicide, limited analysis by specific cancer subgroups, lack of detailed clinical and treatment data, low number of suicide events, retrospective design
Abdel-Rahman, (2019) [25]	45 suicides O/E ratio: 3.70 (95% CI: 2.70–4.95) For patients with PC, socioeconomic adversity is associated with higher suicide risk	Lack of psychiatric data, area-based socioeconomic data rather than individual-level, risk of underreporting or misclassification of suicide, missing data for the PC subgroup, retrospective design, risk of population overlap with other SEER-based studies
Choi et al., (2021) [18]	4 suicides Multivariate analyses in men PC: HR 3.78 (95% CI 1.21–11.78) Multivariate analyses in women PC: not significant	Possible misclassification or underreporting of suicide, missing data for the PC subgroup, limited data on psychosocial factors, low number of suicide events, retrospective design
Ma et al., (2022) [9]	180 suicides Suicide rate: 88.05 per 100,000 person-years SMR: 6.43 (95% CI: 5.49–7.37) Time-stratified ≤2 months: SMR 90.59 (95% CI: 71.39–110.83)—1241.03 suicides per 100,000 person-years—45.5% of suicides 3–12 months: SMR 10.57 (95% CI: 7.82–13.30)—144.83 suicides per 100,000 person-years—31.6% of suicides >12 months: SMR 1.89 (95% CI: 1.35–2.56)—25.87 suicides per 100,000 person-years—22.7% of suicides Multivariate analyses Factors associated with higher suicide risk: male sex (HR 12.80 [95% CI 7.47–21.92], *p* < 0.001), unmarried (HR 1.83 [95% CI 1.21–2.77], *p* = 0.005), divorced/separated/widowed (HR 1.78 [95% CI 1.23–2.57], *p* = 0.002) Factors associated with lower suicide risk: chemotherapy (HR 0.46 [95% CI 0.32–0.65], *p* < 0.001), surgery (HR 0.55 [95% CI 0.34–0.90], *p* = 0.016), black race (HR 0.25 [95% CI 0.11–0.57], *p* = 0.001), diagnosed with pancreatic neuroendocrine tumor (HR 0.49 [95% CI 0.28–0.86], *p* = 0.013)	No data on psychiatric comorbidities, possible misclassification or underreporting of suicide, retrospective design, potential confounding biases, risk of population overlap with other SEER-based studies
Su et al., (2022) [26]	19 suicides Suicide rate: 112.3 per 100,000 person-years SMR: 5.98 (95% CI: 3.81–9.37)	Lack of information on psychiatric history, risk of misclassification of causes of death, missing data for the PC subgroup, inability to accurately analyze patients with ≥3 primary cancers, retrospective design.
Katayama et al., (2023) [19]	55 suicides Suicide rate: 0.76 per 1000 person-years	No data on psychiatric comorbidities, possible misclassification or underreporting of suicide, missing data for the PC subgroup, lack of psychosocial and clinical confounders, low number of suicide events, limited generalizability (Medicare population), retrospective design, risk of population overlap with other SEER-based studies
Hu et al. (2023) [12]	354 suicides SMR: 2.50 (95% CI: 2.24–2.76) Time-stratified ≤2 years: PC was associated with higher suicide risk compared with colorectal cancer (HR adjusted 1.27 [95% CI: 1.12–1.43]) >2 years: PC was associated with lower suicide risk compared with colorectal cancer (HR adjusted 0.25 [95% CI: 0.15–0.40])	Absence of psychiatric/psychosocial variables, possible misclassification of suicide on death certificates, missing data for the PC subgroup, retrospective design
Potter et al. (2023) [27]	20 suicides SMR: 2.08 (95% CI: 1.29–3.19) For patients with PC: higher suicide risk within 1 year after surgery and shorter time from surgery to suicide	Absence of information on psychiatric history, potential misclassification of cause of death, small number of events for some subgroups (including PC), missing data for the PC subgroup, retrospective design, surgical cohort (limited generalizability), risk of population overlap with other SEER-based studies
Michalek et al. (2023) [28]	10 suicides SMR: 1.77 (95% CI: 0.85–3.25) Sex-stratified Men: 9 suicides—SMR 1.84 (95% CI: 0.84–3.50) Women: 1 suicide—SMR 1.29 (95% CI: 0.03–7.20) Time-stratified: Within the first 6 months after cancer diagnosis: 6 suicides—SMR 2.66 (95% CI: 0.98–5.80) 5–10 years after cancer diagnosis: 3 suicides—SMR 8.55 (95% CI: 1.76–24.99)	Absence of psychiatric/psychosocial variables, risk of underreporting or misclassification of suicide, small number of suicide events (low statistical power), missing data for the PC subgroup, retrospective design
Forbes et al. (2024) [29]	14 suicides Incidence rate: 0.33/1000 patient-years Compared to controls, higher suicide risk in patients with PC (HR adjusted 6.85 [95% CI: 2.37–19.78])	Limited psychiatric data, risk of underreporting or misclassification of suicide, small number of suicide events, missing data for the PC subgroup, possible confounding factors missing during multivariate analyses, retrospective design
Wang et al. (2024) [30]	88 suicides Suicide rate: 42.75 per 100,000 person-years SMR: 3.51 (95% CI: 2.81–4.32) Age-stratified Highest suicide risk in patients aged 70–74 years: SMR 5.14 (95% CI 3.10–8.03) Time-stratified: Highest suicide risk during the first 1–4 months after diagnosis: SMR 15.04 (95% CI 11.05–20.00) Multivariate analyses Risk factors for suicide: male sex (HR 12.19 [95% CI 5.47–27.20], *p* < 0.001), absence of chemotherapy (HR 1.65 [95% CI 1.05–2.58], *p* = 0.029), divorced marital status (HR 2.33 [95% CI 1.30–4.19], *p* = 0.005)	Lack of psychiatric or psychosocial data, risk of underreporting or misclassification of suicide, limited details regarding chemotherapy and radiotherapy, possible confounding factors missing during multivariate analyses, retrospective design, risk of population overlap with other SEER-based studies
Kinslow et al. (2024) [31]	295 suicides Suicide rate: 165 per 100,000 person-years SMR: 9.7 (95% CI: 7.8–11.8) Stage-stratified: Localized: SMR 7.3 (95% CI: 3.1–14.3) Distant: SMR 13.6 (95% CI: 10.2–17.9) Regional: SMR 6.2 (95% CI: 3.7–9.6)	Lack of psychiatric or psychosocial data, risk of underreporting or misclassification of suicide, missing data for the PC subgroup, retrospective design, primary analyses limited to the first 6 months after cancer diagnosis, risk of population overlap with other SEER-based studies
Kittel et al., (2025) [20]	18 suicides Suicide rate: 173.9 per 100,000 person-years	Possible misclassification or underreporting of suicide, limited generalizability (veterans’ population, mostly male), missing data for the PC subgroup, misclassification of cancer or treatment data, lack of detailed psychosocial variables, low number of suicide events, retrospective design
Fitzgerald et al., (2025) [8]	19 suicides aIRR (full follow-up): 7.4 (95% CI: 6.3–8.6) aIRR (follow-up restricted to 1 year): 7.5 (95% CI: 5.8–9.8)	No data on psychiatric comorbidities, possible misclassification or underreporting of suicide, missing data for the PC subgroup, potential confounding by unmeasured psychosocial factors, low number of suicide events, retrospective design, main analyses limited to first five years
Gao et al., (2026) [21]	11 suicides SMR: 113.68 (95% CI: 56.67–203.42) Time-stratified ≤1 year: SMR 152.48 (95% CI: 69.58–289.47) 1–5 years: SMR 53.00 (95% CI: 5.95–191.34)	No individual data on psychiatric history or psychosocial factors, possible misclassification or underreporting of suicide, lack of detailed treatment regimens and comorbidity adjustment, low number of suicide events, retrospective design, chemotherapy cohort (limited generalizability), risk of population overlap with other SEER-based studies
Hsiao et al., (2026) [22]	PC among highest suicide risk cancers but with a downward trend Concentration of suicides within the first two years post-diagnosis in patients with PC, followed by a marked decline thereafter After this 2-year post-diagnosis period, a higher suicide risk persists in patients with PC than in the general population.	Ecological design (no causality), no data on psychiatric comorbidities, possible underreporting or misclassification of suicide, missing data for the PC subgroup, long study period with changes in staging or treatment, limited stratified precision (few events), lack of individual psychosocial data, retrospective design
Listabarth et al., (2026) [23]	Higher cumulative suicide incidence for patients with PC in palliative care (*p* = 0.008)	Small number of suicides (low statistical power), potential selection bias (more severe patients in palliative care), possible underreporting or misclassification of suicide, missing data for the PC subgroup, heterogeneous control group, limited adjustment for psychosocial or psychiatric factors

Overall, the available literature indicates that PC is associated with a substantially elevated suicide risk and is consistently identified among the malignancies with the highest relative suicide risk [8,9,10,11,12,13,15,16,17,18,19,20,21,22,23,24,25,26,27,28,29,30,31]. Across studies, SMRs generally ranged from 2.34 to 10.80 [9,11,12,15,17,24,25,26,27,28,29,30,31], O/E ratios from 3.70 to 8.01 [13,25], aIRRs from 7.4 to 7.5 [8], and HRs from 1.27 to 6.85 [12,18,29]. Suicide rates ranged from 42.75 to 321.5 per 100,000 person-years [9,11,15,17,26,30,31], whereas AERs ranged from 1.37 to 4.41 per 10,000 person-years [10]. Despite variability in the magnitude of these estimates, the overall pattern consistently supports a markedly increased risk of suicide among patients with PC across diverse healthcare settings and study designs [8,9,10,11,12,13,15,16,17,18,19,20,21,22,23,24,25,26,27,28,29,30,31].

#### 3.2.2. Time Frame of Suicide Risk After Diagnosis (Table 2)

A robust finding across studies is the clustering of suicide deaths during the early period following cancer diagnosis. This phenomenon appears to be particularly pronounced among patients with PC, with several studies consistently demonstrating that suicide risk peaks during the early post-diagnosis period and subsequently declines over time [9,13,15,17,21,22,28,30,31].

Saad et al. (2019) showed that PC was associated with a markedly elevated suicide risk during the first year following diagnosis, with an O/E ratio of 8.01 (95% CI: 6.29–10.06) [13]. The highest risk was observed during the first six months (O/E ratio 9.46 [95% CI: 7.18–12.23]) and remained elevated, although to a lesser extent, during the subsequent six months (O/E ratio 5.15 [95% CI: 2.94–8.36]) [13]. Similarly, Anderson et al. (2018) reported a particularly elevated suicide risk during the first two months following diagnosis (SMR 9.72 [95% CI: 6.35–14.25]), with persistently increased risk between two months and one year (SMR 7.31 [95% CI: 5.39–9.70]), followed by a progressive decline between one and five years (SMR 2.65 [95% CI: 1.54–4.24]) and a return toward baseline levels after five years (SMR 1.02 [95% CI: 0.12–3.68]) [17].

These findings were further reinforced by Ma et al. (2022), who identified an extremely early peak in suicide risk. Nearly half of all suicides (45.5%) occurred within the first two months following diagnosis, corresponding to an SMR of 90.59 (95% CI: 71.39–110.83) [9]. Although suicide risk subsequently declined, it remained markedly elevated during months 3 to 12 (SMR 10.57 [95% CI: 7.82–13.30]) and persisted beyond one year (SMR 1.89 [95% CI: 1.35–2.56]) [9]. Comparable patterns were observed in South Korea, where Ahn et al. (2010) reported the highest suicide risk during the first year following diagnosis among men (SMR 6.56 [95% CI: 3.96–10.89]), with risk remaining elevated between one and four years (SMR 8.10 [95% CI: 5.17–12.70]) before decreasing substantially after five years (SMR 1.47 [95% CI: 0.37–5.87]) [15]. Among women, the highest suicide risk was likewise observed during the first year following diagnosis (SMR 7.61 [95% CI: 3.17–18.29]) [15].

Several recent studies have provided additional evidence supporting the existence of this early high-risk period. Wang et al. (2024) demonstrated that the greatest suicide risk occurred during the first one to four months following diagnosis, with an SMR of 15.04 (95% CI: 11.05–20.00) [30]. Likewise, Kinslow et al. (2024) reported a suicide rate of 165 per 100,000 person-years and an SMR of 9.7 (95% CI: 7.8–11.8) within the first six months following diagnosis, with particularly elevated risk among patients with distant-stage disease (SMR 13.6 [95% CI: 10.2–17.9]) [31]. In Europe, Michalek et al. (2023) similarly observed that most suicide events occurred shortly after diagnosis, reporting an SMR of 2.66 (95% CI: 0.98–5.80) during the first six months of follow-up [28]. Furthermore, among surgically treated patients, Potter et al. (2023) found that suicide risk was highest during the first year following surgery and that patients with PC experienced a particularly short interval between surgery and suicide compared with patients affected by other malignancies [27].

More recent evidence from specific treatment populations further supports these observations. Gao et al. (2026), in a cohort restricted to patients receiving chemotherapy, reported an exceptionally high SMR of 152.48 (95% CI: 69.58–289.47) during the first year following diagnosis, with persistently elevated suicide risk between one and five years (SMR 53.00 [95% CI: 5.95–191.34]) [21]. Similarly, Hsiao et al. (2026), using nationwide Taiwanese registry data, demonstrated a clear concentration of suicide events during the first two years following diagnosis, followed by a progressive decline thereafter, although suicide risk remained higher than that observed in the general population beyond this period [22]. Additional evidence from the North American Association of Central Cancer Registries indicated that the relative excess suicide risk associated with PC was greatest during the first two years following diagnosis compared with other major malignancies [12].

Importantly, although suicide risk appears to decrease over time, several studies suggest that it does not fully return to baseline levels. Saad et al. (2019) reported persistent excess suicide risk beyond one year following diagnosis (O/E ratio 2.27 [95% CI: 1.36–3.54]) [13], whereas Ma et al. (2022) continued to observe significantly elevated suicide risk beyond 12 months [9]. Similarly, Taiwanese registry data indicated a sustained excess suicide risk despite the progressive decline observed after the initial post-diagnosis period [22].

Overall, the available evidence consistently indicates that suicide risk among patients with PC is greatest during the early post-diagnosis period (with several studies identifying the greatest excess risk within the first two months following diagnosis) and remains elevated throughout the first year before progressively declining over time [9,13,15,17,21,22,28,30,31]. In large population-based studies, risk estimates during the early post-diagnosis period, variably defined from the first 2 months to the first year after diagnosis, generally ranged from SMRs of 6.56 to 90.59 [9,15,17,28,30,31], O/E ratios from 5.15 to 11.61 [13], and an aIRR of 7.5 [8]. Although the magnitude and duration of this excess risk vary across studies, the temporal pattern is remarkably consistent across countries, healthcare systems, and study designs, with the earliest post-diagnosis period representing the peak of vulnerability [9,12,13,15,17,21,22,28,30,31]. These findings suggest that the weeks and months immediately following a PC diagnosis constitute a critical window for psychosocial assessment, suicide risk screening, and the implementation of targeted supportive interventions.

#### 3.2.3. Factors Associated with Suicide Risk (Table 2)

Male sex consistently emerged as one of the strongest and most reproducible predictors of suicide risk among patients with PC [9,11,15,18,24,28,30]. Turaga et al. (2011) reported substantially higher odds of suicide among men than among women (OR 13.5 [95% CI: 3.2–56.9]) [11]. Similarly, Ma et al. (2022) identified male sex as an independent predictor of suicide risk in multivariable analyses (HR 12.80 [95% CI: 7.47–21.92]) [9]. More recently, Wang et al. (2024) confirmed this association, reporting a significantly increased suicide risk among men (HR 12.19 [95% CI: 5.47–27.20]) [30]. Consistent findings were observed in Asian populations. In South Korea, Ahn et al. (2010) reported substantially higher suicide risk among men (SMR 6.01 [95% CI: 4.33–8.33]) than among women (SMR 2.76 [95% CI: 1.15–6.63]) [15], whereas Choi et al. (2021) demonstrated a significantly increased suicide risk among men with PC (HR 3.78 [95% CI: 1.21–11.78]), while no statistically significant association was observed among women [18]. Similar sex-specific patterns were reported in registry-based studies from Lithuania and Poland, where suicide risk was consistently higher among male patients [24,28].

Social determinants, particularly marital status, also appear to play an important role in suicide risk. Turaga et al. (2011) found that being married was associated with a significantly lower suicide risk among men (OR 0.3 [95% CI: 0.1–0.6]) [11]. Likewise, Ma et al. (2022) reported increased suicide risk among unmarried patients (HR 1.83 [95% CI: 1.21–2.77]) and among those who were divorced, separated, or widowed (HR 1.78 [95% CI: 1.23–2.57]) [9]. Similar findings were reported by Wang et al. (2024), who identified divorced marital status as an independent predictor of suicide risk (HR 2.33 [95% CI: 1.30–4.19]) [30]. Furthermore, Abdel-Rahman (2019) demonstrated that socioeconomic deprivation was associated with an increased suicide risk among patients with PC, highlighting the broader influence of social vulnerability on suicide outcomes [25].

Disease severity also appears to contribute substantially to suicide risk. Saad et al. (2019) reported a markedly higher suicide risk among patients with distant-stage disease (O/E ratio 11.61 [95% CI: 8.47–15.53]) than among those with localized or regional disease (O/E ratio 4.80 [95% CI: 3.01–7.27]) [13]. Similarly, Kinslow et al. (2024), evaluating suicides occurring within the first six months following diagnosis, found the highest suicide risk among patients with metastatic disease (SMR 13.6 [95% CI: 10.2–17.9]), exceeding that observed among patients with localized disease (SMR 7.3 [95% CI: 3.1–14.3]) or regional disease (SMR 6.2 [95% CI: 3.7–9.6]) [31]. These findings support the hypothesis that poor prognosis, substantial symptom burden, and awareness of limited life expectancy may contribute to heightened psychological distress among patients with advanced disease [13,31].

Regarding treatment-related factors, findings were more heterogeneous. Ma et al. (2022) reported that chemotherapy (HR 0.46 [95% CI: 0.32–0.65]) and surgery (HR 0.55 [95% CI: 0.34–0.90]) were independently associated with lower suicide risk [9]. Similarly, Wang et al. (2024) found that the absence of chemotherapy was associated with an increased suicide risk (HR 1.65 [95% CI: 1.05–2.58]) [30]. In contrast, Turaga et al. (2011) reported that surgery was associated with a higher suicide risk among men (OR 2.5 [95% CI: 1.0–6.4) [11]. Furthermore, Potter et al. (2023), in a cohort restricted to surgically treated patients, observed that suicide risk was highest during the first year following surgery and that patients with PC experienced a particularly short interval between surgery and suicide compared with patients affected by other malignancies [27]. Collectively, these findings suggest that associations between treatment and suicide risk may vary according to the timing of assessment, patient selection, disease stage, and survivorship status [9,11,27,30].

Additional patient-related characteristics have also been associated with suicide risk. In the SEER analysis conducted by Ma et al. (2022), Black race was associated with a lower suicide risk than White race (HR 0.25 [95% CI: 0.11–0.57]), while patients diagnosed with pancreatic neuroendocrine tumors exhibited a lower suicide risk than those with pancreatic adenocarcinoma (HR 0.49 [95% CI: 0.28–0.86]) [9]. Age may also influence suicide risk. Wang et al. (2024) reported the highest relative suicide risk among patients aged 70–74 years (SMR 5.14 [95% CI: 3.10–8.03]) [30].

Findings from specific high-risk populations further support the broader literature. Kittel et al. (2025), using Veterans Health Administration data, reported a suicide rate of 173.9 per 100,000 person-years among patients with PC, one of the highest rates observed across all cancer types [20]. Likewise, Listabarth et al. (2026) demonstrated a significantly higher cumulative incidence of suicide among patients with PC receiving palliative care than among registry-based controls, underscoring the vulnerability of individuals with advanced disease and substantial symptom burden [23]. Moreover, Forbes et al. (2024) reported that patients with PC had an almost sevenfold higher suicide risk than matched cancer-free controls (adjusted HR 6.85 [95% CI: 2.37–19.78]), indicating that the excess risk persists even after adjustment for several demographic characteristics [29].

Overall, the available evidence suggests that suicide risk among patients with PC is driven by multiple interacting factors. However, only a limited number of studies have formally examined these factors using multivariable analyses. Across these studies, male sex emerged as the most consistent independent predictor of suicide [9,18,30]. Marital status was also associated with suicide risk, with unmarried, divorced, separated, or widowed individuals exhibiting a higher risk in several analyses. Nevertheless, these findings were largely derived from SEER-based cohorts with partially overlapping populations and should therefore be interpreted cautiously [9,11,30]. Additional evidence suggests that socioeconomic disadvantage may further increase vulnerability to suicide [25]. Treatment-related factors were investigated less consistently. Chemotherapy was associated with lower suicide risk in two multivariable analyses based on partially overlapping SEER-derived populations [9,30], whereas the evidence regarding surgery was inconsistent, with one study reporting a favorable association [9] and another, restricted to men, reporting an increased risk [11]. Although stage-stratified analyses suggested higher suicide risks among patients with advanced disease [13,31], the available evidence does not consistently support disease stage as an independent predictor of suicide. More broadly, descriptive and subgroup analyses indicate that patients with poorer prognostic characteristics and greater social vulnerability may be at particularly high risk, although the evidence remains limited and heterogeneous [13,20,23,25,29,30,31]. Collectively, these findings highlight the importance of incorporating systematic psychosocial assessment, suicide risk screening, and targeted preventive interventions into routine pancreatic cancer care.

### 3.3. Quality Assessment and Risk of Bias of the Selected Studies (Table 3)

All included studies in this systematic review were observational in nature and therefore were classified as level 4 evidence (Grade C recommendation) according to the ANAES/HAS hierarchy [32]. In addition, risk of bias was assessed using the ROBINS-E tool (Risk Of Bias In Non-randomized Studies of Exposures) for each exposure-outcome association. Most studies were judged to be at high risk of bias, while a smaller number raised some concerns. The main source of bias was residual confounding, reflecting the observational nature of the included studies and the limited availability of important suicide-related factors. Concerns related to post-exposure interventions and outcome measurement were also common. By contrast, exposure measurement, participant selection, and missing data were generally judged to be at low risk of bias. Detailed ROBINS-E assessments for each study and exposure-outcome association are presented in Table 3.

**Table 3:** Evaluation of biases according to the ROBINS-E tool.

Studies	D1	D2	D3	D4	D5	D6	D7	Global Risk	Exposure	Outcome
Ahn et al., (2010) [15]	High risk	Low risk	Low risk	Some concerns	Low risk	Some concerns	Some concerns	High risk	Cancer diagnosis	Suicide mortality
Turaga et al., (2011) [11]	High risk	Low risk	Low risk	Some concerns	Low risk	Some concerns	Some concerns	High risk	Cancer diagnosis	Suicide mortality
Nasseri et al., (2012) [16]	High risk	Low risk	Low risk	Some concerns	Low risk	Some concerns	Some concerns	High risk	Cancer diagnosis	Suicide mortality
Kaceniene et al., (2017) [24]	High risk	Low risk	Low risk	Some concerns	Low risk	Some concerns	Some concerns	High risk	Cancer diagnosis	Suicide mortality
Anderson et al., (2018) [17]	High risk	Low risk	Low risk	Some concerns	Low risk	Some concerns	Some concerns	High risk	Cancer diagnosis	Suicide mortality
Saad et al., (2019) [13]	High risk	Low risk	Low risk	Some concerns	Low risk	Some concerns	Some concerns	High risk	Cancer diagnosis	Suicide mortality
Henson et al., (2019) [10]	High risk	Low risk	Low risk	Some concerns	Low risk	Some concerns	Some concerns	High risk	Cancer diagnosis	Suicide mortality
Abdel-Rahman, (2019) [25]	High risk	Low risk	Low risk	Some concerns	Low risk	Some concerns	Some concerns	High risk	Cancer diagnosis	Suicide mortality
Choi et al., (2021) [18]	Some concerns	Low risk	Low risk	Some concerns	Low risk	Some concerns	Some concerns	Some concerns	Cancer diagnosis	Suicide mortality
Ma et al., (2022) [9]	High risk	Low risk	Low risk	Some concerns	Low risk	Some concerns	Some concerns	High risk	Cancer diagnosis	Suicide mortality
Su et al., (2022) [26]	High risk	Low risk	Low risk	Some concerns	Low risk	Some concerns	Some concerns	High risk	Cancer diagnosis	Suicide mortality
Katayama et al., (2023) [19]	Some concerns	Low risk	Low risk	Some concerns	Low risk	Some concerns	Some concerns	Some concerns	Cancer diagnosis	Suicide mortality
Hu et al. (2023) [12]	High risk	Low risk	Low risk	Some concerns	Low risk	Some concerns	Some concerns	High risk	Cancer diagnosis	Suicide mortality
Potter et al. (2023) [27]	High risk	Low risk	Low risk	Some concerns	Low risk	Some concerns	Some concerns	High risk	Cancer surgery	Suicide mortality
Michalek et al. (2023) [28]	High risk	Low risk	Low risk	Some concerns	Low risk	Some concerns	Some concerns	High risk	Cancer diagnosis	Suicide mortality
Forbes et al. (2024) [29]	Some concerns	Low risk	Low risk	Some concerns	Low risk	Low risk	Some concerns	Some concerns	Cancer survivorship	Suicide mortality
Wang et al. (2024) [30]	High risk	Low risk	Low risk	Some concerns	Low risk	Some concerns	Some concerns	High risk	Cancer diagnosis	Suicide mortality
Kinslow et al. (2024) [31]	High risk	Low risk	Low risk	Some concerns	Low risk	Some concerns	Some concerns	High risk	Cancer diagnosis	Suicide mortality
Kittel et al., (2025) [20]	Some concerns	Low risk	Low risk	Some concerns	Low risk	Low risk	Some concerns	Some concerns	Cancer survivorship	Suicide mortality
Fitzgerald et al., (2025) [8]	Low risk	Low risk	Low risk	Some concerns	Low risk	Low risk	Some concerns	Some concerns	Cancer diagnosis	Suicide mortality
Gao et al., (2026) [21]	High risk	Low risk	Some concerns	High risk	Some concerns	Some concerns	Some concerns	High risk	Chemotherapy	Suicide mortality
Hsiao et al., (2026) [22]	High risk	Low risk	Low risk	Some concerns	Low risk	Some concerns	Some concerns	High risk	Cancer diagnosis	Suicide mortality
Listabarth et al., (2026) [23]	High risk	Low risk	Some concerns	High risk	Low risk	Some concerns	Some concerns	High risk	Palliative care	Suicide mortality

D1: Bias due to confounding; D2. Bias arising from measurement of the exposure; D3: Bias in selection of participants into the study; D4: Bias due to post-exposure interventions; D5: Bias due to missing data; D6: Bias arising from measurement of the outcome; D7: Bias in selection of the reported result.

## 4. Discussion

### 4.1. Epidemiology of Suicide Among Patients with PC

This systematic review demonstrates that suicide represents a clinically significant and likely underrecognized cause of excess mortality among patients with PC. Despite differences in study designs, outcome definitions, healthcare systems, and analytical approaches, the available evidence consistently indicates that individuals diagnosed with PC experience a substantially elevated suicide risk compared with the general population. Most studies reported relative risks that were several-fold higher than expected, placing PC among the malignancies most strongly associated with suicide risk.

One of the most robust findings emerging from this review is the temporal distribution of suicide risk. Across multiple countries and datasets from diverse healthcare systems, suicide risk was particularly elevated during the early post-diagnosis period (with several studies identifying the greatest excess risk within the first two months following diagnosis), remained elevated throughout the first year and gradually declined thereafter [9,13,15,17,21,22,28,30,31]. Although the magnitude of this excess risk varied across studies, a remarkably consistent temporal pattern was observed, suggesting that the period immediately following diagnosis constitutes a particularly vulnerable phase [9,12,13,15,17,21,22,28,30,31]. Importantly, the available evidence also indicates that suicide risk does not completely resolve over time and may remain elevated beyond the first year following diagnosis, particularly among patients with advanced disease or those receiving palliative care [9,13,15,17,21,22,23,28].

Nevertheless, the interpretation of these temporal trends should take into account the exceptionally high disease-specific mortality associated with PC. Given the short survival observed in many patients, competing cancer mortality substantially reduces the population remaining at risk of suicide over time. Consequently, survivors at later time points represent a progressively selected subgroup characterized by more favorable prognostic and clinical features. The apparent decline in suicide risk observed during follow-up may therefore reflect not only a reduction in psychological vulnerability but also the effects of competing mortality and survivorship selection. This methodological consideration is particularly relevant in PC, where high early mortality markedly influences the distribution of person-time at risk and the interpretation of long-term suicide patterns. However, the consistent observation of a pronounced excess suicide risk immediately after diagnosis across multiple independent cohorts suggests that this early peak is unlikely to be explained solely by survivorship-related mechanisms.

This temporal pattern is clinically plausible. A diagnosis of PC frequently confronts patients with a poor prognosis, limited life expectancy, the prospect of intensive treatments associated with substantial adverse effects, and the possibility of rapid physical decline [34,35]. Consequently, the immediate post-diagnosis period may be characterized by profound psychological distress, disruption of future life plans, and difficulties adapting to the new reality imposed by the disease [9,10,11,13,15,17,21,22,27,30,31]. However, the persistence of elevated suicide risk beyond the initial post-diagnosis phase suggests that psychological vulnerability is not confined to the time of diagnosis and may persist throughout the disease trajectory [9,13,21,22,23].

Taken together, these findings support the recognition of suicide prevention as an integral component of comprehensive pancreatic cancer care. They further suggest that suicide risk assessment should not be restricted to a single time point but should instead be incorporated into longitudinal oncological and supportive care pathways, with particular attention to the period immediately following diagnosis and other clinically vulnerable phases of the disease course.

### 4.2. Epidemiology of Suicide in PC Compared with Other Cancers

A notable finding of this review is that the association between PC and suicide risk appears stronger than that observed for most other malignancies. Across studies directly comparing different cancer types, PC was repeatedly identified as one of the malignancies associated with the highest relative suicide risk [9,11,15,17,25,30,31]. This observation was remarkably consistent across diverse populations, healthcare systems, and methodological approaches, suggesting that it is unlikely to be explained solely by cultural, geographic, or study-specific factors [8,9,10,15,17,18,19,22,24,25,28,29,30,31]. Collectively, these findings support the notion that the elevated suicide risk associated with PC represents a robust and reproducible epidemiological phenomenon rather than a context-specific observation.

Although an increased suicide risk has been reported across a wide range of cancer diagnoses, the pattern observed in PC appears particularly striking for two reasons. First, the magnitude of the excess risk is among the highest reported in oncology [8,9,10,15,17,18,19,22,24,25,29,30,31]. Second, this excess risk emerges very early following diagnosis, often within the first weeks or months [9,13,17,22,28,30,31]. Taken together, these characteristics distinguish PC from many other malignancies in which suicide risk is either less pronounced or more evenly distributed throughout the disease course.

The mechanisms underlying this distinctive epidemiological profile are likely multifactorial. The combination of poor prognosis, frequent presentation with advanced disease, substantial symptom burden, and profound psychological distress may create conditions in which vulnerability to suicide is amplified compared with that observed in other malignancies. Furthermore, the abrupt confrontation with a life-threatening diagnosis and limited therapeutic prospects may further contribute to psychological distress during the early stages of the disease trajectory. Consequently, PC may represent a particularly informative model for understanding the complex interplay between oncological, psychological, and social determinants of suicide [8,9,10,13,15,17,18,19,22,24,25,29,30,31].

### 4.3. Potential Hypotheses Explaining the Increased Suicide Risk in Patients with PC

Several hypotheses derived from the existing literature may be advanced to explain the elevated suicide risk in patients with PC observed in this systematic review. These potential mechanisms are likely complex and interrelated. While some reflect challenges commonly encountered across cancer diagnoses, others may be more specific to PC and could help explain the particularly high vulnerability observed in this patient population. Nevertheless, the available evidence remains largely indirect, and these explanations should be interpreted as plausible hypotheses rather than established mechanisms.

#### 4.3.1. General Hypotheses Associated with Suicide Risk in Oncology

Several mechanisms potentially linking cancer and suicide are not specific to PC and have been described across a wide range of malignancies. A cancer diagnosis frequently represents a major psychological stressor, associated with uncertainty, fear, disruption of personal and professional life, and concerns regarding future health and wellbeing [36]. For many patients, the diagnosis initiates a period of psychological adjustment during which emotional resilience may be profoundly challenged [29].

Depression is one of the most important contributors to suicidal behaviors in oncology. Patients with cancer experience depressive symptoms more frequently than individuals in the general population, and depression has consistently been associated with suicidal ideation, suicide attempts, and suicide risk [37]. Beyond major depressive disorder, other forms of psychological distress, including anxiety, hopelessness, demoralization, fear of the future, and social isolation, may also contribute to vulnerability to suicide [38]. These emotional responses may be further exacerbated by concerns related to prognosis, symptom burden, treatment effectiveness, and the potential loss of independence and autonomy.

Biological mechanisms may further interact with these psychological processes. Systemic inflammation, a hallmark of many malignancies, has increasingly been implicated in the pathophysiology of both depression and suicidality. Pro-inflammatory cytokines may influence neurotransmitter pathways, neuroendocrine regulation, and neural circuits involved in mood regulation and stress responses [39,40]. Consequently, cancer-related inflammation may contribute not only to physical symptoms but also to neuropsychiatric manifestations that increase susceptibility to suicide [41].

These psychological and biological processes frequently interact with the burden imposed by cancer treatment and its impact on daily life. Anticancer therapies may be associated with substantial adverse effects, functional impairment, and diminished quality of life [42,43]. At the same time, patients often face considerable social and economic challenges, including loss of independence, reduced autonomy, financial hardship, occupational disruptions, and progressive social isolation [44,45]. Collectively, these factors provide a biopsychosocial framework that helps explain why cancer is consistently associated with an increased suicide risk across diverse oncological settings.

#### 4.3.2. Specific Hypotheses Associated with Suicide Risk in PC

Although many determinants of suicide are shared across malignancies, several characteristics of PC may help explain why the association with suicide risk appears particularly strong in this disease.

The most apparent factor is its exceptionally poor prognosis. Despite recent therapeutic advances, the 5-year survival rate for PC remains below 12% across all stages combined [46]. Compared with several common malignancies, including breast, prostate, and colorectal cancers, PC continues to be associated with limited long-term survival, particularly among patients diagnosed with advanced disease [47,48]. Awareness of this prognosis may contribute to feelings of hopelessness, loss of purpose, and existential distress, all of which are recognized factors associated with increased suicide risk and psychological vulnerability [49].

The manner in which PC is typically diagnosed may further intensify this psychological burden. Because effective population-based screening strategies remain unavailable, many patients receive the diagnosis at an advanced stage of disease and often with little prior warning [50]. This abrupt transition from perceived health to a life-threatening illness may leave limited time for psychological adaptation and coping [51,52], rendering the disclosure of the diagnosis particularly distressing compared with malignancies that follow a more gradual clinical trajectory [53,54].

Beyond the initial shock of diagnosis, the subsequent clinical course of PC may also contribute substantially to suicide risk. Patients frequently experience severe pain, cachexia, fatigue, nutritional impairment, and rapid functional decline [55,56], often to a greater extent and over a shorter time frame than that observed in many other solid tumors [57,58]. These symptoms can profoundly impair quality of life, independence, and daily functioning, thereby increasing psychological distress [55,56,57,58,59]. The close relationship between symptom burden and emotional suffering may partly explain the elevated suicide risk observed among patients with advanced disease [60,61]. In addition, PC appears to have a particularly strong association with depression. Several studies have reported depressive symptoms preceding the diagnosis of PC, leading to the hypothesis that tumor-related biological mechanisms may directly influence mood regulation [62,63].

Although the underlying mechanisms remain incompletely understood, the pronounced inflammatory state associated with PC has been proposed as a potential contributor to the neuropsychiatric manifestations observed in this disease [64,65]. Chronic inflammation may amplify biological pathways implicated in depression and suicidality, thereby exacerbating psychological vulnerability. Furthermore, the occurrence of depressive symptoms before cancer diagnosis in some patients has led to the hypothesis that paraneoplastic or tumor-related processes may directly affect mood regulation [66,67]. Collectively, these mechanisms may contribute to the particularly strong relationship between PC, depression, and suicide risk [68].

Therapeutic constraints may represent an additional source of psychological vulnerability. Although substantial advances have recently been achieved in systemic therapies, treatment options for PC remain more limited than those available for several other common malignancies, particularly in the metastatic setting [69,70,71]. Consequently, some patients may perceive fewer opportunities for durable disease control or long-term survival, potentially contributing to feelings of helplessness, uncertainty, and despair [72].

Overall, the increased suicide risk associated with PC is most appropriately understood within a biopsychosocial framework in which general cancer-related determinants of suicide interact with disease-specific characteristics of PC. These include poor prognosis, diagnosis at an advanced stage, abrupt confrontation with a life-threatening illness, substantial symptom burden, accelerated physical decline, biological influences on mood regulation, and limited therapeutic prospects. Together, these factors may create a uniquely vulnerable context that contributes to the markedly elevated suicide risk observed among patients with PC.

### 4.4. Potential Strategies to Reduce the Suicide Risk in Patients with PC

The findings of this review have important clinical implications. Given the magnitude of the suicide risk observed among patients with PC, the available epidemiological evidence supports increased clinical awareness of psychological distress and suicide vulnerability in this population. However, the studies included in this review did not evaluate whether specific screening strategies or interventions reduce suicide mortality.

Particular attention should be directed toward the period immediately following diagnosis, which consistently emerged as the phase of greatest vulnerability. Routine assessment of psychological distress and depressive symptoms using validated instruments, such as the Distress Thermometer, the Patient Health Questionnaire-9 (PHQ-9), and the Hospital Anxiety and Depression Scale (HADS), may help identify patients experiencing substantial emotional burden [73,74]. However, distress or depression screening should not be regarded as equivalent to a formal suicide-risk assessment, as these instruments were not designed specifically to predict suicide. Patients presenting with significant psychological distress, depressive symptoms, or suicidal ideation may require more comprehensive clinical evaluation and, when appropriate, referral to specialized mental health services [75]. Furthermore, such assessments could reasonably be considered throughout follow-up, as psychological needs and levels of distress may evolve during the disease trajectory [75].

The identification of high-risk subgroups may further help guide preventive efforts. Across the available literature, male sex and unmarried status emerged as the factors most consistently associated with increased suicide risk, whereas socioeconomic disadvantage and advanced disease may potentially contribute to vulnerability in some patient populations [11,17]. Targeted monitoring and tailored supportive interventions for these vulnerable populations may enhance the effectiveness of suicide prevention strategies.

However, screening alone is unlikely to be sufficient unless accompanied by appropriate clinical support. Although direct evidence demonstrating a reduction in suicide mortality is currently lacking, multidisciplinary care may represent a reasonable approach for addressing the complex psychological, social, and physical needs of patients with PC [76,77,78]. Close collaboration among oncology, psycho-oncology, psychiatry, psychology, and palliative care teams may facilitate earlier recognition of psychological distress and provide a more comprehensive approach to symptom management [76,77,78]. While these integrated care models have been associated with improvements in quality of life and supportive care outcomes [76,77,78], their specific impact on suicide prevention in patients with PC remains to be established.

Although some observational studies have reported lower suicide rates among patients receiving active anticancer treatment, potentially owing to improved symptom control, maintenance of hope, and more frequent interactions with healthcare professionals [9], these findings should be interpreted with caution. Treatment allocation is strongly influenced by disease stage, prognosis, functional status, and baseline patient vulnerability, all of which may confound the observed associations [11]. Nevertheless, these observations underscore the importance of maintaining therapeutic engagement and regular clinical contact with vulnerable patients throughout the disease course [9].

Ultimately, the findings of this review support the need for heightened awareness of suicide vulnerability and psychological distress throughout the PC trajectory. Systematic psychological assessment, timely access to mental health services, multidisciplinary management, and targeted support for vulnerable patients represent plausible strategies to address this burden, but their effectiveness in reducing suicide mortality has not been specifically demonstrated in the studies reviewed. Prospective studies are therefore needed to determine whether such approaches improve suicide-related outcomes. Nevertheless, the consistently elevated suicide risk observed across studies supports consideration of suicide prevention and psychosocial support as components of comprehensive pancreatic cancer care, alongside oncological treatment, symptom management, and supportive care.

### 4.5. Limitations

Several limitations should be considered when interpreting the findings of this systematic review. First, the literature search was restricted to the PubMed-Medline and Scopus databases. While this approach may have resulted in the omission of a limited number of potentially relevant studies indexed exclusively in other databases, the risk of missing a substantial proportion of the available evidence is likely low, given that PubMed-Medline and Scopus together provide very broad coverage of the biomedical, medical, and health sciences literature. Beyond limitations related to the review process itself, the included studies presented important methodological constraints. All studies were observational in nature and predominantly retrospective, relying largely on administrative databases and population-based cancer registries. Although these data sources provide access to large populations and enable the investigation of rare outcomes such as suicide, they remain susceptible to residual confounding and generally offer limited clinical, psychosocial, and psychiatric information. In addition, several studies were derived from the SEER database and likely included partially or substantially overlapping patient populations. Turaga et al. (2011) analyzed patients diagnosed between 1992 and 2005 [11] and therefore likely shared a proportion of participants with subsequent SEER-based studies. The most notable overlap concerns Anderson et al. (2018) and Saad et al. (2019) [13,17], which analyzed the same cohort of 119,194 patients with PC diagnosed between 2000 and 2014, although they addressed different research questions. Additional substantial overlap is likely among Abdel-Rahman (2019), Ma et al. (2022), Wang et al. (2024), and Kinslow et al. (2024) [9,25,30,31], which examined large SEER-derived cohorts covering broadly overlapping periods between 2000 and 2019. Furthermore, Katayama et al. (2023) (restricted to Medicare beneficiaries), Potter et al. (2023) (restricted to surgically treated patients) and Gao et al. (2026) (restricted to chemotherapy-treated patients) likely represent nested subsets of the broader SEER population rather than independent cohorts [19,21,27]. Consequently, these studies should be considered complementary analyses of overlapping populations rather than entirely independent sources of evidence. Nevertheless, consistent findings from several independent non-SEER data sources [8,10,15,16,18,22,23,28,29] support the robustness of the observed association between PC and increased suicide risk. This convergence of evidence across distinct populations and healthcare systems strengthens the validity of the overall findings despite the potential overlap among SEER-based cohorts.

Several important determinants of suicide risk, including psychiatric history, severity of depression, substance use disorders, social support, and previous suicide attempts, were infrequently available and therefore could not be adequately accounted for in most analyses. The ascertainment of suicide outcomes also warrants consideration. In most studies, suicide cases were identified through death certificates, cancer registries, or administrative coding systems. Consequently, misclassification cannot be excluded, particularly when suicides are recorded as accidental deaths or deaths of undetermined intent. Such misclassification may have resulted in an underestimation of the true burden of suicide among patients with PC [79]. Another important limitation relates to the availability of detailed cancer-specific information. Key clinical variables, including tumor stage, treatment trajectories, disease progression, symptom burden, and treatment response, were inconsistently reported across studies. This limited the ability to perform robust stratified analyses and restricted a more comprehensive understanding of the respective contributions of disease severity, treatment exposure, and clinical evolution to suicide risk. Furthermore, substantial heterogeneity was observed across the included studies. Differences in study populations, outcome definitions, follow-up periods, healthcare systems, and statistical methodologies complicated direct comparisons. Although exploratory quantitative syntheses and subgroup analyses were performed, the very high level of between-study heterogeneity limited the interpretability of pooled estimates. Based on these elements, these analyses were not incorporated into the main manuscript and are instead presented in the Supplementary Data S2, where they should be interpreted with caution. The external validity of the findings should also be considered, as most of the available evidence originated from high-income countries, particularly the United States. Variations in healthcare accessibility, availability of oncology and mental health services, socioeconomic conditions, cultural attitudes toward suicide, and patterns of cancer care may limit the generalizability of these findings to other geographic and healthcare settings.

Nevertheless, these limitations should be interpreted in the context of the overall consistency of the available evidence. Despite variations in study design, populations, healthcare systems, outcome definitions, and analytical approaches, the direction of the association remained remarkably consistent across studies. The reproducibility of findings across independent cohorts and multiple countries strengthens confidence in the existence of a meaningful association between PC and increased suicide risk. However, the observational nature of the available evidence and the substantial heterogeneity between studies limit causal inference and make it difficult to determine the precise magnitude of the excess risk. Future prospective studies incorporating detailed psychiatric, psychosocial, biological, and oncological variables are needed to better elucidate the mechanisms underlying this association and to identify patients who may derive the greatest benefit from targeted suicide prevention strategies.

## 5. Conclusions

PC is associated with a substantial excess suicide risk, making suicide an important yet often underrecognized component of the overall disease burden. The available evidence consistently indicates that this risk is among the highest reported across malignancies and is particularly concentrated during the first year following diagnosis (with several studies identifying the greatest excess risk within the early post-diagnosis period), followed by a gradual decline thereafter. Male sex emerged as the most consistently demonstrated risk factor for suicide. Associations with unmarried status were observed in several studies and are clinically plausible, but the supporting evidence derives largely from partially overlapping SEER-based cohorts and is therefore less robust. Additional evidence suggests a potential contribution of social vulnerability and adverse prognostic characteristics. The mechanisms underlying this association are likely multifactorial and involve the combined effects of poor prognosis, high symptom burden, psychological distress, biological alterations that may affect mood regulation, and the broader social and functional consequences of the disease. Although some studies have suggested a potential association between active anticancer treatment and lower suicide risk, the available evidence remains insufficient to establish a causal relationship. Overall, these findings highlight the importance of awareness of suicide vulnerability and psychological distress among patients with PC and support consideration of psychosocial assessment, suicide risk evaluation, and timely access to mental health support within comprehensive pancreatic cancer care. Future prospective studies incorporating detailed psychiatric, psychosocial, and clinical data are needed to better characterize high-risk patients, clarify the mechanisms underlying this association, and inform the development of effective suicide prevention strategies in this particularly vulnerable population.

## Data Availability

All data, codes, and other materials are available in the manuscript and tables.

## Supplementary Materials

The following supporting information can be downloaded at https://www.mdpi.com/article/10.3390/diseases14090346/s1, Data S1: Classification of evidence levels and recommendation grades (ANAES/HAS) used to assess the quality of included studies; Data S2: Methodology and results of exploratory quantitative analyses and subgroup analyses evaluating suicide risk in patients with pancreatic cancer [8,9,10,11,12,13,15,17,21,24,25,26,27,28,30,31].
