# Supplementary material for: Suicide Risk and Associated Factors in Patients with Pancreatic Cancer: A Systematic Review
Source: Diseases. 2026 Sep 18;14(9):346. doi: 10.3390/diseases14090346 (PMC13605502; doi:10.3390/diseases14090346)
Supplement: Supplementary file 1 [file diseases-14-00346-s001.zip › diseases-4476925-supplementary.pdf]

Supplementary Data – S1

Grade of recommendations	Level of scientific evidence provided by the literature
Grade A<br>Established scientific evidence	Level 1<br>- High-powered randomized controlled trials<br>- Meta-analyses of randomized controlled trials<br>- Decision analyses based on well-conducted studies
Grade B<br>Scientific presumption	Level 2<br>- Low-powered randomized controlled trials<br>- Well-conducted non-randomized comparative studies<br>- Cohort studies
Grade C<br>Low level of scientific evidence	Level 3<br>- Case-control studies
	Level 4<br>- Comparative studies with significant bias<br>- Retrospective studies<br>- Case series<br>- Descriptive epidemiological studies (cross-sectional, longitudinal)

<u>Supplementary Data – S2</u>

1/Methodology

1.1/Quantitative Synthesis

A quantitative synthesis was performed whenever studies reported a relative measure of suicide risk compared with the general population. Standardized mortality ratios (SMRs), observed-to-expected (O/E) ratios, rate ratios (RRs), and adjusted incidence rate ratios (aIRRs) were considered for inclusion in an exploratory quantitative synthesis. Because these measures all reflect relative comparisons between suicide occurrence among patients with PC and a reference population, they were considered sufficiently similar to provide an approximate quantitative summary of the available evidence. However, these measures are not strictly equivalent from a methodological perspective and differ in their statistical construction, underlying assumptions, and comparator populations. Consequently, pooled estimates should be interpreted as exploratory indicators of the direction and magnitude of the association rather than precise effect estimates.

Studies included in the quantitative synthesis were those reporting SMRs, O/E ratios, suicide rate ratios, or aIRRs in patients with PC, including Ahn et *al*. (2010), Turaga et *al*. (2011), Kaceniene et *al*. (2017), Anderson et *al*. (2018), Saad et *al*. (2019), Henson et *al*. (2019), Abdel-Rahman (2019), Ma et *al*. (2022), Su et *al*. (2022), Hu et *al*. (2023), Potter

et *al*. (2023), Michalek et *al*. (2023), Wang et *al*. (2024), Kinslow et *al*. (2024), Fitzgerald et *al*. (2025), and Gao et *al*. (2026) [8-13,15,17,21,24-28,30,31].

For each study, the natural logarithm of the reported effect estimate and its standard error were calculated from the published 95% confidence intervals using standard meta-analytic methods. A random-effects model based on the DerSimonian and Laird approach was applied to account for anticipated between-study heterogeneity arising from differences in study populations, healthcare systems, clinical settings, follow-up duration, and methods of suicide ascertainment.

Statistical heterogeneity was assessed using Cochran's Q test and the I^2 statistic, with values exceeding 75% considered indicative of substantial heterogeneity. Sensitivity analyses were conducted by excluding studies identified as potential outliers. Prespecified subgroup analyses were performed according to geographical region (North America, Europe, and Asia), publication period (2010-2019 versus 2020-2026), and type of effect estimate (SMR versus other relative risk measures).

1.2/Temporal Analysis of Suicide Risk

In addition to the primary meta-analysis, an exploratory temporal subgroup analysis was conducted to evaluate changes in suicide risk according to the time elapsed since PC diagnosis. Studies reporting risk estimates during predefined post-diagnosis intervals were identified and reviewed. When multiple time-specific estimates were reported within a study, the earliest available risk estimate following diagnosis was

selected for quantitative synthesis in order to assess the period of maximum vulnerability. This approach was based on the consistent observation that suicide risk appears to peak shortly after cancer diagnosis.

Studies contributing to the temporal analysis included Ahn et *al*. (2010), Anderson et *al*. (2018), Saad et *al*. (2019), Ma et *al*. (2022), Wang et *al*. (2024), Kinslow et *al*. (2024), and Gao et *al*. (2026), all of which reported suicide risk during the first year following diagnosis or during even earlier follow-up intervals [9,13,15,17,21,30,31]. Reported periods ranged from the first two months to the first twelve months after diagnosis.

A separate random-effects meta-analysis was performed using these early post-diagnosis estimates to obtain a pooled measure of suicide risk during the highest-risk period following PC diagnosis. Because exceptionally high estimates were reported by Ma et *al*. (2022) and Gao et *al*. (2026) [9,21], an additional sensitivity analysis excluding these studies was prespecified to evaluate the robustness of the temporal association and to assess the influence of extreme effect estimates on the pooled result.

Beyond quantitative pooling, a narrative temporal synthesis was conducted to describe longitudinal changes in suicide risk throughout follow-up. Studies reporting multiple time intervals were examined to identify trends in risk evolution after diagnosis, including the transition from the immediate post-diagnosis period to long-term survivorship. Particular attention was given to studies reporting suicide risk beyond one year after diagnosis in order to assess whether excess risk persisted over time.

All pooled effect estimates were reported as relative risks with corresponding 95% confidence intervals, and statistical significance was inferred when confidence intervals did not include unity. Results from the primary analysis, sensitivity analyses, subgroup analyses, and temporal analyses were interpreted collectively to characterize the magnitude, consistency, and timing of suicide risk among patients with PC.

2/Results

2.1/Included Studies

Sixteen studies reporting relative estimates of suicide risk among patients with PC compared with the general population were eligible for quantitative synthesis. These studies were conducted across North America, Europe, and Asia and included large population-based cohorts derived from national cancer registries and administrative healthcare databases.

2.2/Primary Analysis

The pooled random-effects analysis demonstrated a significantly increased suicide risk among patients with PC compared with the general population. The pooled relative risk estimate was 5.58 (95% CI 4.04-7.72). Substantial heterogeneity was observed across studies (I^2=96.9%).

Because one study (Gao et *al*., 2026) reported an exceptionally high effect estimate and was restricted to patients receiving chemotherapy [21], a sensitivity analysis was

subsequently performed excluding this study. The pooled estimate remained statistically significant and clinically meaningful, yielding a pooled relative risk of 4.73 (95% CI 3.49-6.40), thereby confirming the robustness of the overall findings. Heterogeneity remained substantial (I^2=96.5%).

2.3/Subgroup Analyses

Subgroup analyses demonstrated a consistently elevated suicide risk across all examined categories.

When stratified by geographical region, the pooled relative risk was 6.52 (95% CI 4.29-9.92) among North American studies and 3.51 (95% CI 1.84-6.68) among European studies. The only eligible Asian study reported an SMR of 6.01 (95% CI 4.33-8.33).

When stratified according to publication period, studies published between 2010 and 2019 yielded a pooled relative risk of 5.35 (95% CI 3.73-7.67), whereas studies published from 2020 onward yielded a pooled relative risk of 5.88 (95% CI 3.64-9.50).

Restriction of the analysis to studies reporting SMRs produced a pooled relative risk of 5.48 (95% CI 3.69-8.12), confirming consistency across methodological approaches. The persistence of a markedly elevated pooled estimate when the analysis was restricted to studies reporting SMRs suggests that the overall findings are not solely explained by the combination of different effect measures and supports the robustness of the observed association.

Studies reporting alternative risk measures (O/E ratios or aIRRs) demonstrated a pooled estimate of 6.13 (95% CI 4.13-9.11). The similarity of pooled estimates obtained among studies reporting SMRs and those reporting alternative relative measures supports the overall consistency of the observed association despite differences in effect metrics. Nevertheless, comparisons across these groups should be interpreted cautiously because the underlying measures are not strictly interchangeable.

2.4/Temporal Analysis of Suicide Risk

A dedicated temporal subgroup analysis was conducted using the earliest risk estimate reported after PC diagnosis. Seven studies provided sufficient data for quantitative synthesis of the immediate post-diagnosis period.

The pooled random-effects analysis demonstrated that suicide risk during the first months following diagnosis was markedly higher than that observed in the overall study population, with a pooled relative risk of 19.54 (95% CI 8.35-45.73). Heterogeneity was substantial (I^2=98.1%).

Because Ma et *al*. (2022) and Gao et *al*. (2026) reported exceptionally elevated estimates [9,21], an additional sensitivity analysis was performed excluding these outliers. The pooled relative risk remained significantly increased at 10.10 (95% CI 8.09-12.61), with a marked reduction in heterogeneity (I^2=60.4%).

Across individual studies, suicide risk was consistently highest immediately after diagnosis and declined progressively over time. Anderson et *al*. (2018) reported a

decrease from an SMR of 9.72 within the first two months to 2.65 between one- and five-years following diagnosis [17]. Similarly, Ma et *al*. (2022) observed a decline from an SMR of 90.59 during the first two months to 10.57 during months 3-12 and 1.89 beyond the first year [9]. Ahn et *al*. (2010) also demonstrated substantially lower suicide risk after five years of follow-up compared with the first year after diagnosis [15].

These findings indicate that the excess suicide risk observed among patients with PC is not constant over time but follows a distinct temporal pattern characterized by a sharp peak immediately following diagnosis and a gradual decline thereafter.

3/Summary of Meta-Analysis Results

Analyses	Studies (n)	Pooled RR	95% CI	I^2 (%)
Primary analysis (all studies)	16	5.58	4.04-7.72	96.9
Sensitivity analysis (excluding Gao et *al*.)	15	4.73	3.49-6.40	96.5
North America	11	6.52	4.29-9.92	97.7
Europe	4	3.51	1.84-6.68	91.2
Asia	1	6.01	4.33-8.33	N/A
Publications (2010-2019)	7	5.35	3.73-7.67	92.1
Publications (2020-2026)	9	5.88	3.64-9.50	97.8
Studies reporting SMRs	13	5.48	3.69-8.12	97.3
O/E ratios or aIRRs	3	6.13	4.13-9.11	89.2
Early post-diagnosis period (≤12 months)	7	19.54	8.35-45.73	98.1
Early post-diagnosis period (sensitivity analysis)	5	10.10	8.09-12.61	60.4

4/Quantitative Conclusion

Overall, the available evidence suggests that PC is associated with an increased suicide risk, particularly during the first year following diagnosis. However, the interpretation of these findings is substantially limited by the very high heterogeneity across studies, reflecting important differences in study populations, healthcare settings, methodological approaches, and outcome ascertainment. Furthermore, the quantitative synthesis combined several related but non-identical effect measures, including SMRs, O/E ratios, suicide rate ratios, and aIRRs. Although all these measures evaluate relative suicide risk in comparison with a reference population, differences in their statistical definitions, derivation methods, and comparator populations may have contributed to additional heterogeneity and imprecision in pooled estimates. Consequently, pooled estimates should be regarded as indicative of a general trend rather than precise measures of effect. The principal value of the quantitative synthesis lies in demonstrating the consistency and direction of the association across studies rather than providing an exact quantification of the magnitude of excess suicide risk. Further standardized research is needed to better define the magnitude and timing of suicide risk in patients with PC.
